# Medical Items

**Published:** 1892-08

**Authors:** 


### *MEDICAL ITEMS.*

DR. M. A. PURSE, originally of Savannah, has located in Atlanta.

DR. R. R. KIME will confine his practice hereafter to obstetrics and diseases of women.

THE Illinois Board of Health does not accept a death certificate bearing the word "heart-failure."

DR. J. A. CHILDS and Dr. George Brown, of Atlanta, are pursuing special courses at the Post-Graduate School, New York.

THE University of Georgia at its last commencement conferred the degree of doctor of laws upon Dr. Joseph Jones, of New Orleans. The honor was worthily bestowed.

DR. CHAS. D. ROY sailed for Europe in July. Abroad he will confine his studies to diseases of the eye, ear, nose and throat. Dr. Roy will be gone about one year.

AT Mrs. Agnew's request, Dr. J. Howe Adams, of Philadelphia, is preparing a biography of the late distinguished Dr. D. Hayes Agnew. Dr. Adams will be pleased to receive any interesting data from the friends and former pupils of Dr. Agnew, for which credit will be given in the biography.

THE Mississippi Valley Medical Association will hold its eighteenth annual session at Cincinnati, Wednesday, Thursday and Friday, October 12th, 13th and 14th, 1892. A large attendance and á valuable programme are expected. Chas. A. L. Reed, President, E. S. McKee, Secretary, Cincinnati.

DR. KEEN, of Philadelphia, recently did an amputation at the hip joint for a rapidly growing sarcoma, the patient being five months pregnant. Recovery was rapid and pregnancy uninterrupted.

---

DR. H. R. HARRIS has been elected Professor of Chemistry in the Southern Medical Callege, and Dr. Ralph E. Smith becomes Lecturer on Rectal and Genito-Urinary diseases in the Atlanta Medical College.

---

DR. S. LATIMER PHILLIPS and Dr. T. H. Bloodworth, both of Savannah, sailed in July for Europe. Dr. Phillips will return in the fall. Dr. Bloodworth will spend one year in study in foreign medical centers. We hope these gentlemen will kindly let our readers hear from them while abroad.

---

THE fourth annual meeting of the Tri-State Medical Society of Alabama, Georgia and Tennessee will be held in Chattanooga October 25th, 26th and 27th. Those wishing to join the society, or to read papers, or to exhibit specimens, should address the secretary, Dr. Frank Trester Smith, Chattanooga, Tenn.

---

A SHREWD observer records (*Revue de Medicine*) the case of a boy who became inoculated with pulmonary tuberculosis by bed-bugs. It appears that these latter had formerly occupied the bed with two of the boy's brothers, both of whom had died of the disease. Several of the animals were captured and careful experiments revealed the presence of large numbers of tubercle bacilli concealed about their persons. Thus it seems that the pestilence that walketh in darkness, though not provided with wings, has a deadly way of " getting there all the same."

THOSE who believe in the ancient origin of syphilis will be interested in the paper of Proksch (*Archives für Dermatologie*), who claims to have discovered in some old Egyptian papyrus additional evidence both as to the existence and treatment of syphilis at a very remote period. The papyrus describes a disease known as " uxedu," which affected the arms, the eyes, the mouth, the bones, etc. The author identifies this as modern syphilis—the same yesterday, to-day and forever.

---

KEELEY'S gold-mine (not the bichloride) at Dwight, Illinois, is now being worked at the rate of about $17,500 per week. The New York *Sun*, of recent date, contained a letter from Dr. A. R. Stephens, of Herricksville, Pennsylvania, about the Keeley business, of which the following is an extract:

"I subjected the remedy (Keeley's) to a careful chemical analysis, and found it consisted of a preparation of gentian, cinchona and a trace of opium, without any gold in any shape, unless represented in the price! I consider the remedy—taking that sent to me as a fair sample—a perfect humbug, and have kept a sample of it, which I am ready to exhibit to any chemist or professional man at any time."

Not long since the Board of Health of Illinois withdrew from Dr. Keeley his licénse to practice medicine on the ground that he had violated the spirit of the code. For political reasons the license was restored to him by Governor Fifer.

---

WE prs ent our acknowledgments to Dr. Emory Lanphear, of Kansas City, Mo., for report of a laparotomy done under cocaine. Patient, aged fifty-two, was suffering from cancer of neck. Swallowing impossible. Gastrostomy was done, after injecting one-half drachm of a four per cent. cocaine solution along proposed line of incision. Operation lasted twenty-two minutes, was without discomfort and was not followed by shock.

